# Cyclotron resonance in the high mobility GaAs/AlGaAs 2D electron system over the microwave, mm-wave, and terahertz- bands

**DOI:** 10.1038/s41598-019-39186-2

**Published:** 2019-02-20

**Authors:** A. Kriisa, R. L. Samaraweera, M. S. Heimbeck, H. O. Everitt, C. Reichl, W. Wegscheider, R. G. Mani

**Affiliations:** 1 Georgia State University, Dept. of Physics and Astronomy, Atlanta, 30303 USA; 2 Army Aviation and Missile RD&E Center, Redstone Arsenal, 35898 USA; 3 Duke University, Dept. of Physics, Durham, 27708 USA; 4 ETH-Zürich, Laboratorium für Festkörperphysik, Zürich, 8093 Switzerland

## Abstract

The reflected microwave power from the photo-excited high mobility GaAs/AlGaAs 2D device has been measured over the wide frequency band spanning from 30 to 330 GHz simultaneously along with diagonal magnetoresistance as a function of the magnetic field. Easily distinguishable resonances in the reflected power signal are observed at the same magnetic fields as a reduced amplitude in the Shubnikov-de Haas (SdH) oscillations of the diagonal magnetoresistance. The reflection resonances with concurrent amplitude reduction in SdH oscillations are correlated with cyclotron resonance induced by microwave, mm-wave, and terahertz photoexcitation. The magnetoplasma effect was also investigated. The results suggest a finite frequency zero-magnetic-field intercept, providing an estimate for the plasma frequency. The experimentally measured plasma frequency appears to be somewhat lower than the estimated plasma frequency for these Hall bars. The results, in sum, are consistent with an effective mass ratio of m*/m = 0.067, the standard value, even in these high mobility GaAs/AlGaAs devices, at very large filling factors. Preliminary findings from this article have been published as conference proceedings, see Kriisa, A., *et al*., *J. of Phys. Conf. Ser*. **864**, 012057 (2017).

## Introduction

Two dimensional electron systems have constituted a remarkable arena for the development of condensed matter physics over, approximately, the last forty years^1^. More recently, the study of novel emergent phenomena in the photo-excited transport properties of such low-dimensional systems have been a focus of interest^2–76^. Emergence is found in systems that are far from equilibrium. Associated phenomena are characterized by complex behaviors that arise out of a multiplicity of basic interactions, and they can exhibit remarkable collective and cooperative aspects. It turns out that a high quality two-dimensional electron system based on GaAs/AlGaAs heterostructures subjected to modest constant microwave/mm-wave/terahertz photo-excitation at low temperature, for example, develops novel steady state non-equilibrium effects. Such non-equilibrium effects can be manifested in the transport properties of the photo-excited system as temperature and radiation-frequency dependent oscillatory changes in the electrical resistance as a function of the magnetic field, and the associated photo-excited oscillatory resistance minima evolve into novel radiation-induced zero-resistance states - zero-resistance states which are characterized by an absence of Hall quantization in a two dimensional electron system^3–31,33–43,45,46^. The observations of photo-excited zero-resistance states and associated magnetoresistance oscillations has also brought with it new interest in the microwave/mm-wave/terahertz reflection, absorption, and transmission properties of the GaAs/AlGaAs 2D electron systems (2DES) at low magnetic fields, including the remote detection of the cyclotron resonance (CR), and the correlation of CR with magnetotransport^10^.

It is well known that the parabolic energy bands in 2D electronic systems, at finite magnetic fields, *B*, are split into quantized Landau levels, separated by cyclotron energy *ℏω*_*c*_ = $\hslash \frac{eB}{{m}^{\ast }}$. Here, *ℏ* is the reduced Planck constant, *ω*_*c*_ is the cyclotron frequency, m^*^ is the effective mass, and *e* is the electron charge. Photo-excitation of this system with electromagnetic radiation leads to absorption of energy, i.e., to cyclotron resonance, if the photon energy equals the cyclotron energy, *hf* = *ℏω*_*c*_, where *f* is the photon frequency^77^. Classically, one obtains the picture that the electrons undergo circular motion perpendicular to the magnetic field, in the plane of the 2DES, as the Lorentz force causes centripetal acceleration. The angular frequency of this circular motion *ω* = 2*πf* = *qB*/m^*^ is independent of the radius of the circular orbit and the electron velocity. Thus, when a circularly polarized electric field at frequency *f*, is applied at the electron cyclotron frequency, i.e., 2*πf* = *ω*_*c*_, with the same sense of rotation as the electron orbit, the electron accelerates under the influence of the electric field of the radiation, which increases the electron velocity, leading to energy gain and a larger orbit radius without changing the frequency of cyclotron motion, until collisions lead to energy relaxation. Here, note that circular polarization in a sense opposite to the rotation of the electrons would not allow for the resonant acceleration and absorption of energy from the electromagnetic field, i.e., cyclotron resonance. Thus, cyclotron resonance under fixed orientation circularly polarized photo-excitation is observable only for one orientation of the magnetic field, the cyclotron resonance active orientation - the orientation for which the sense of circular polarization is the same as the cyclotron rotation of electrons. Cyclotron resonance is often viewed within the context of the so-called “Kohn’s theorem,” which states that electron-electron interactions do not modify the cyclotron frequency in a system characterized by translational invariance and parabolic dispersion relation^78^. However, interactions in a disordered system are thought to modify the cyclotron frequency^79^.

Typically, cyclotron resonance studies are carried out with circularly polarized radiation. However, cyclotron resonance is also observable under linearly polarized photo-excitation since linearly polarized radiation can be decomposed into a sum of cyclotron resonance active- and inactive- components. Since, after such decomposition, an active component will exist for both the normal and reverse magnetic field orientation, cyclotron resonance becomes possible and observable for both directions of the magnetic field under linearly polarized photoexcitation, unlike for circularly polarized photo-excitation. From the law for conservation of energy, the total incident irradiation energy that reaches the sample should equal the sum of the energy absorbed in the sample, the energy reflected back from the sample, and the energy transmitted through the sample. Thus, at cyclotron resonance, the resonant coupling between the 2DES and the electromagnetic radiation should be registered in the absorbed, reflected, and transmitted power signals. Here we examine cyclotron resonance in the electromagnetic wave signal reflected back from a high mobilitiy GaAs/AlGaAs 2DES. In addition to collecting the reflected power signal as a function of the magnetic field at fixed photo-excitation energies, we simultaneously measured the diagonal magnetoresistance, *R*_*xx*_, in order to examine Shubnikov-de Haas oscillations in *R*_*xx*_ for concurrent signatures of cyclotron resonance. By utilizing the knowledge that cyclotron resonance is accompanied by resonant electron heating, we here take advantage of the high sensitivity of Shubnikov de Haas (SdH) oscillations to small increases in the electron temperature to identify the signature of cyclotron resonance in the SdH lineshape^6,17,44^. The observed cyclotron resonance in the magnetoreflection signal is compared with the cyclotron resonance signature in the Shubnikov de Haas oscillations, and the effective mass extracted from the two methods are reported and compared. The magnetoplasma effect was also investigated by plotting *f*^2^ vs. *B*^2^. The results suggested a finite frequency zero-magnetic-field intercept, providing an estimate for the plasma frequency. The results are consistent with an effective mass ratio of m^*^/*m* = 0.067, the standard value, even in these high mobility GaAs/AlGaAs devices.

## Results

**Figure 1:**
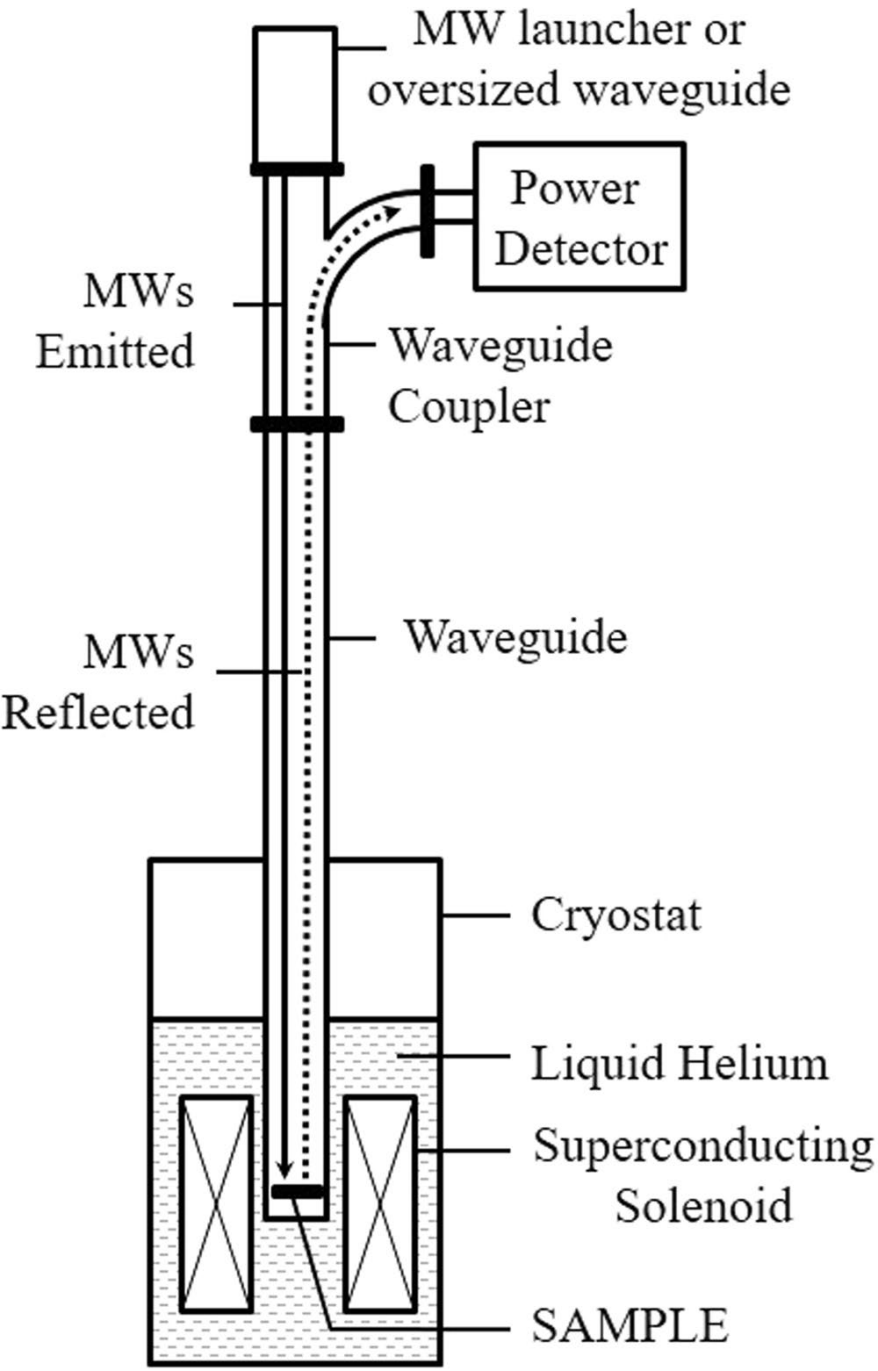
The experimental setup with a sample of 2D GaAs/AlGaAs submerged in the liquid helium cryostat within a superconducting solenoid. Solid line with an arrow indicates the path of the in-bound electromagnetic waves, while the dotted line heading in the opposite direction indicates the waves reflected back from the sample. Reflected waves are directed to the power detector with the waveguide coupler and read out with a power meter.

Figure 1 shows the experimental configuration where the samples are immersed in a liquid helium cryostat within a superconducting solenoid and irradiated with linearly polarized electromagnetic waves, through a waveguide.

**Figure 2:**
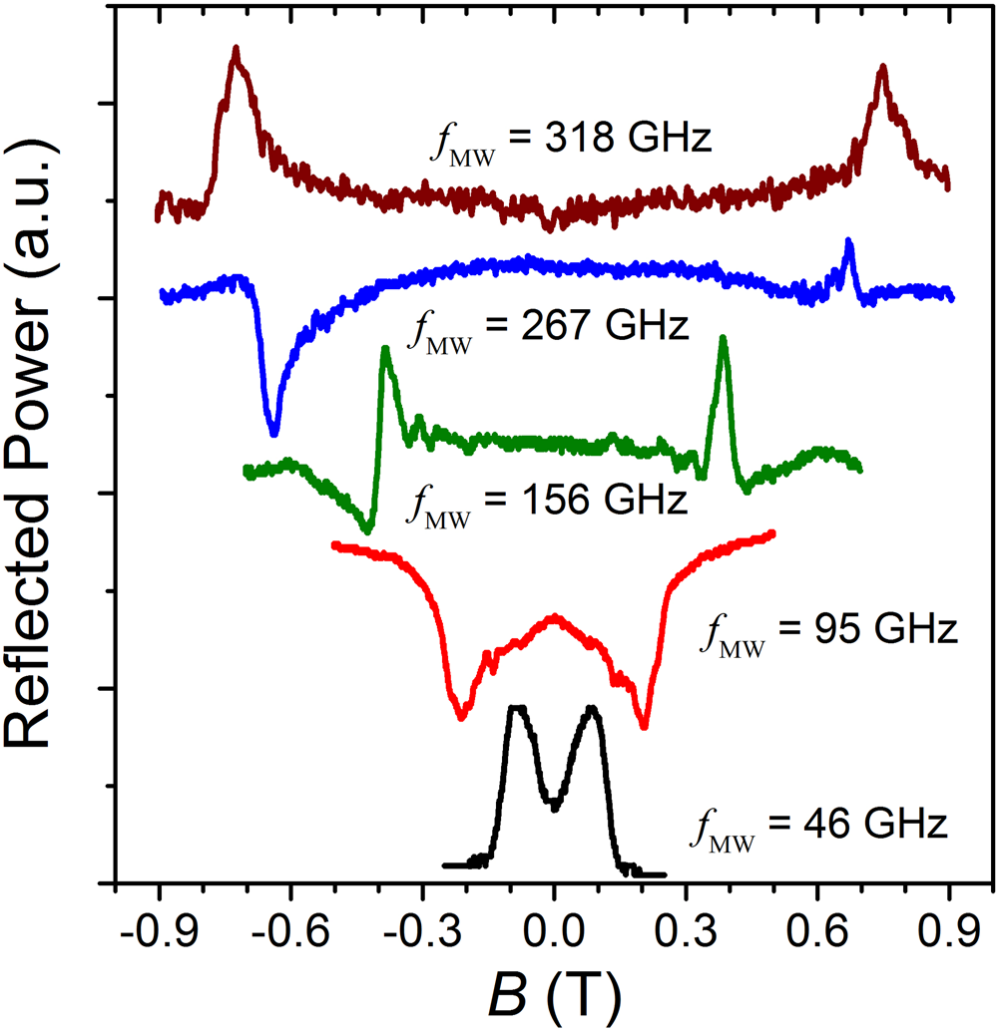
The reflected power signals as a function of the magnetic field measured with different mm-wave and terahertz electromagnetic wave frequencies, *f*, show strong resonanes in the GaAs/AlGaAs 2DES heterostructures at temperature *T* = 1.7 K.

**Figure 3:**
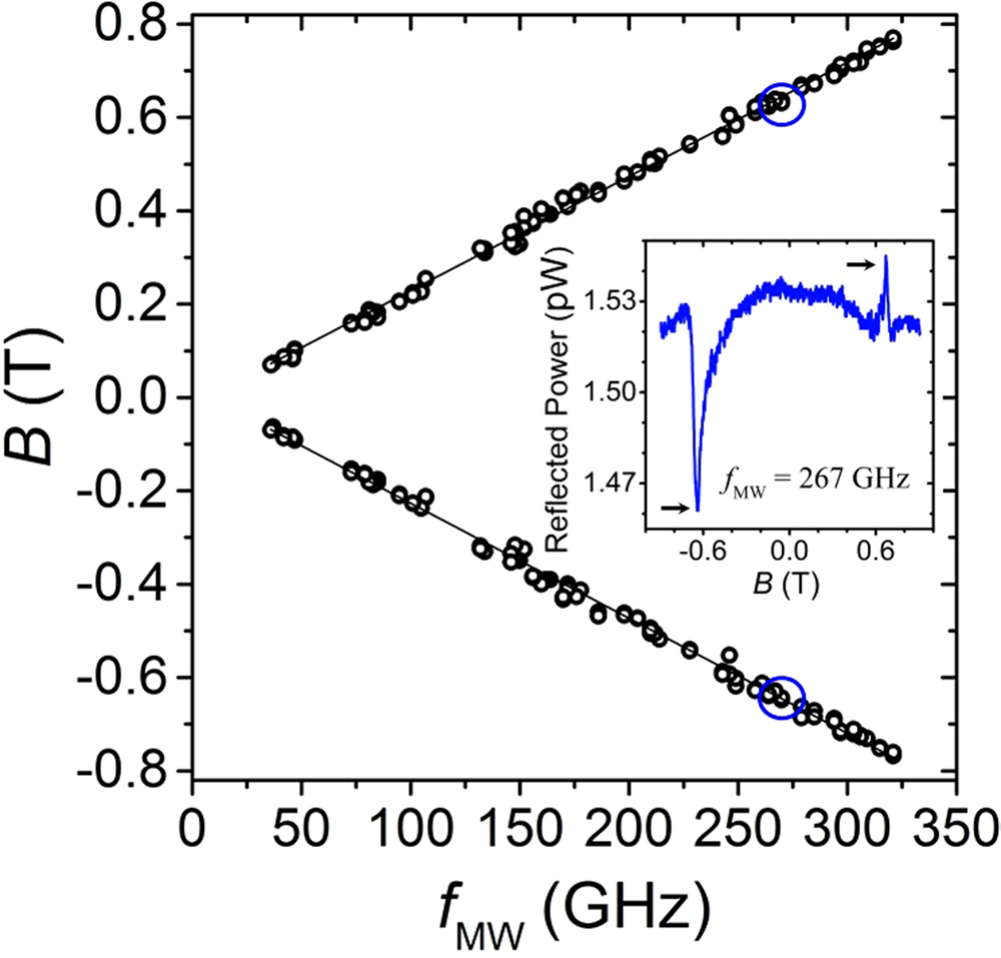
Reflected power resonance positions in magnetic field as a function of microwave frequency f in the GaAs/AlGaAs 2DES. Black lines are linear fits through the data points. The slope values, found from linear fits through both the positive and negative magnetic field data, agree to within experimental error. Inset: arrows point to peaks observed in reflected power measured at frequency *f* = 267 GHz and correspond to data points located inside blue circles in main graph.

Figure 2 shows a selection of reflected power signals measured at different frequencies in a GaAs/AlGaAs 2DES. As linearly polarized electromagnetic waves were used in these experiments, a resonance signal, attributable to cyclotron resonance, is seen on both sides, positive and negative, of the magnetic field axis. Easily distinguishable peaks/valleys, as seen in Fig. 2, appear at each measured frequency, from 36 GHz to 330 GHz. Resonance peaks/valleys in the reflected power signal were not observed for frequencies below 36 GHz. For each irradiation frequency, the measurement was conducted two times, once by sweeping the magnetic field in one direction, then a second time by sweeping in the opposite direction. Power signals were found to be identical for both sweeping directions. As seen in Fig. 2, the shape, symmetry, and even the direction of the tip of the resonance peak (up or down) varied from frequency to frequency. However, the location of resonance peak steadily shifted to higher magnetic field values with increasing frequency. Occasionally, the resonance orientation changed direction even within the same irradiation frequency over different sides of magnetic field, as for example for *f* = 267 GHz. The changing symmetry of the lineshape with the frequency appears reminiscent of the signature of a Fano-type resonance, where interference between a continuum and a resonant scattering process brings about an asymmetric line-shape^80^. Further studies are being carried out to identify the origin of the lineshape observed in this reflection signal. The physical origin of the observed resonance peaks/valleys in the reflected power signal becomes clearer when the peak/valley positions in magnetic field are plotted as a function of microwave frequency *f*, as shown in Fig. 3. Resonance positions at positive and at negative magnetic field are plotted separately, so as to ascertain their symmetrical location along the *B*-axis. As would be expected for cyclotron resonance, resonance positions in magnetic field increase approximately linearly with increasing frequency throughout the examined frequency range. The location of the resonances in reflected power is found to be same on both sides of the magnetic field, as the slopes of linear fits through the data, *dB*/*df* = 2.44 ± 0.02 mT/GHz for positive side and *dB*/*df* = −2.46 ± 0.01 mT/GHz for negative side of magnetic field, agree to within experimental error. A feature of interest in Fig. 2 is that the resonances are rather broad in the magnetic field scale. This suggests a relatively short single particle lifetime for the carriers^81^.

**Figure 4:**
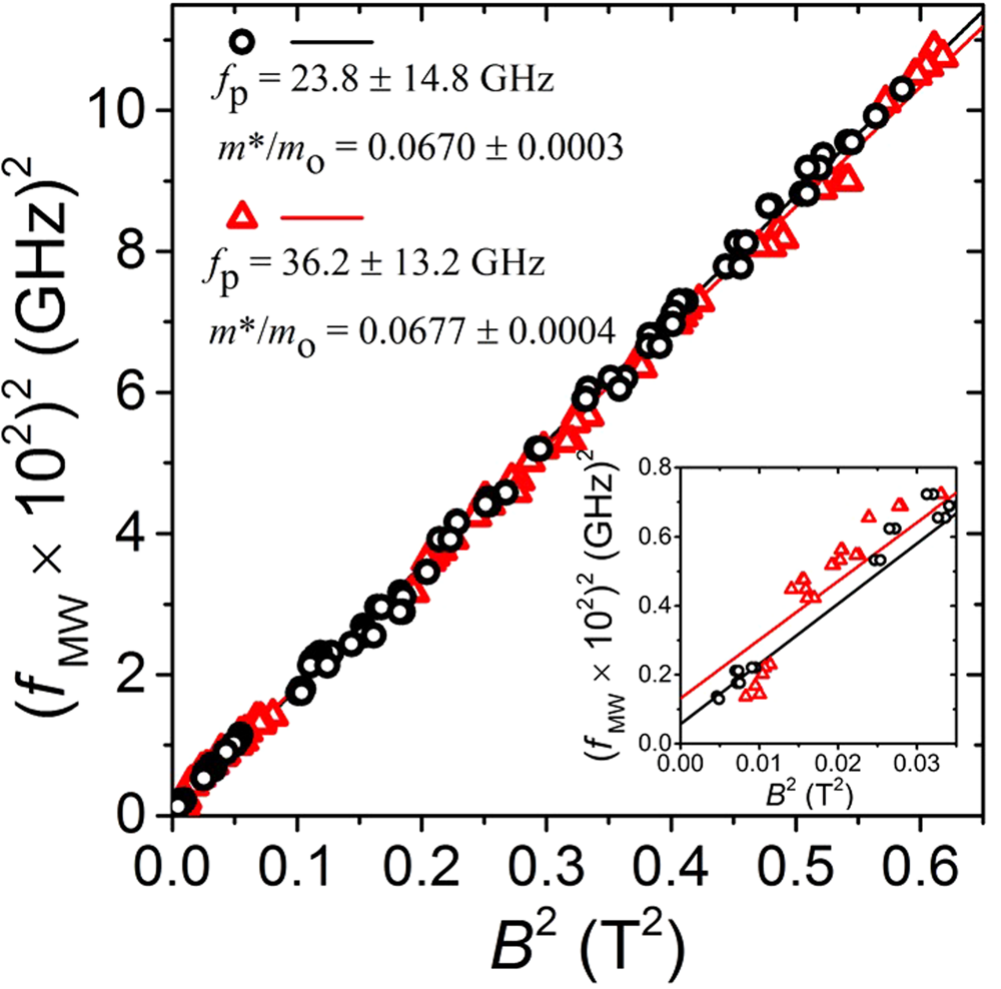
This figure compares the resonance peak locations in the reflected power measured in two different samples, plotted as *f*^2^ vs *B*^2^. Black circles and red triangles correspond to data measured in samples with density *n*_*s*_ ≈ 2.4 × 10^11^ cm^−2^ (data replotted from Fig. 3) and *n*_*s*_ ≈ 3.3 × 10^11^ cm^−2^, respectively. Effective mass ratios, m^*^/*m*, are found from slopes of linear fit through the data, while plasmon frequencies, *f*_*p*_, are found from the square root of the intercept. Inset: close-up of main graph near the origin.

In 2D electron systems, additional electrodynamic effects like plasma oscillations can noticeably shift the measured resonance response with respect to the cyclotron frequency^51,59^. To find whether resonance peaks in this experiment were influenced by electron plasma oscillations, the peak positions shown in Fig. 3 are replotted along with data measured from the second sample on a quadratic scale, i.e. *f*^2^ vs *B*^2^ in Fig. 4 (black circles and red triangles, respectively). Each data point shown in Fig. 4 represents an absolute average of peak positions over the negative and the positive magnetic field sides. Hybridization of cyclotron resonance frequency, f_*CR*_, and plasmon frequency, *f*_*p*_, under magnetic fields perpendicular to 2DES, is described by the magnetoplasmon frequency, *f*_*mp*_ = $\sqrt{{({f}_{CR})}^{2}+{({f}_{p})}^{2}}$, where electrons in the plasma oscillate with frequency, *f*_*p*_ = $\sqrt{\frac{{n}_{s}{e}^{2}}{2{\varepsilon }_{eff}{\varepsilon }_{o}{m}^{\ast }}k}$, where n_*s*_ is the electron density, e is the electron charge, *ε*_*eff*_ is the effective dielectric constant (for GaAs *ε*_*eff*_ = 6.9)^51^, *ε*_*o*_ is the permittivity of free space, m* is the effective mass, and k is the plasmon wave vector. Often, the simple assumption of plasma confinement *k* ≈ *π*/*W* is made, where width of sample, *W*, is half the plasmon wavelength^51^. With this assumption, we find the estimate *f*_*p*_(*n*_*s*_ ≈ 2.4 × 10^11^ cm^−2^) = 57.3 GHz and, *f*_*p*_(*n*_*s*_ ≈ 3.3 × 10^11^ cm^−2^) = 67.2 GHz for the two samples. These calculated values are much higher than the plasmon frequencies *f*_*p*_(*n*_*s*_ ≈ 2.4 × 10^11^ cm^−2^) = 23.8 ± 14.8 GHz (black) and *f*_*p*_(*n*_*s*_ ≈ 3.3 × 10^11^ cm^−2^) = 36.2 ± 13.2 GHz (black) extracted directly from the experimental data, i.e. from square root of the intercept of linear fit through the data in Fig. 4. It seems that the theoretical model used here does not adequately describe the experimental findings, as resonance peaks in the reflected power signal are observed below 57.3 GHz for both samples, all the way down to 36 GHz. We do not believe that the difference between theoretical and experimentally found values of *f*_*p*_ could be explained with the presence of retardation effects, as the dimensionless retardation parameter *α* ≈ 0.29 $\sqrt{{n}_{s}{\mathrm{[10}}^{11}c{m}^{-2}]\times W[mm]}$ ≈ 0.2 for our samples^59^. Retardation effects start influencing magnetotransport only once retardation parameter approaches unity, i.e. when the velocity of 2D plasmons approach the velocity of light^59^.

In Fig. 4, the slopes of the linear fit *d*(*B*)^2^/*d*(*f*_*MW*_)^2^ = (*e*/2πm*)^2^, give the effective mass m* = (0.0670 ± 0.0003)_*mo*_ (black) and m* = (0.0677 ± 0.0004)*m*_*o*_ (red), where *m*_*o*_ is the free electron mass. These values for m* agree, to within experimental error, with the most frequently quoted m* = 0.067 m_*o*_ for GaAs/AlGaAs devices. However, the observed effective mass ratio in these measurements exceeds the value extracted from the study of the radiation-induced magnetoresistance oscillations in similar materials^5^.

**Figure 5:**
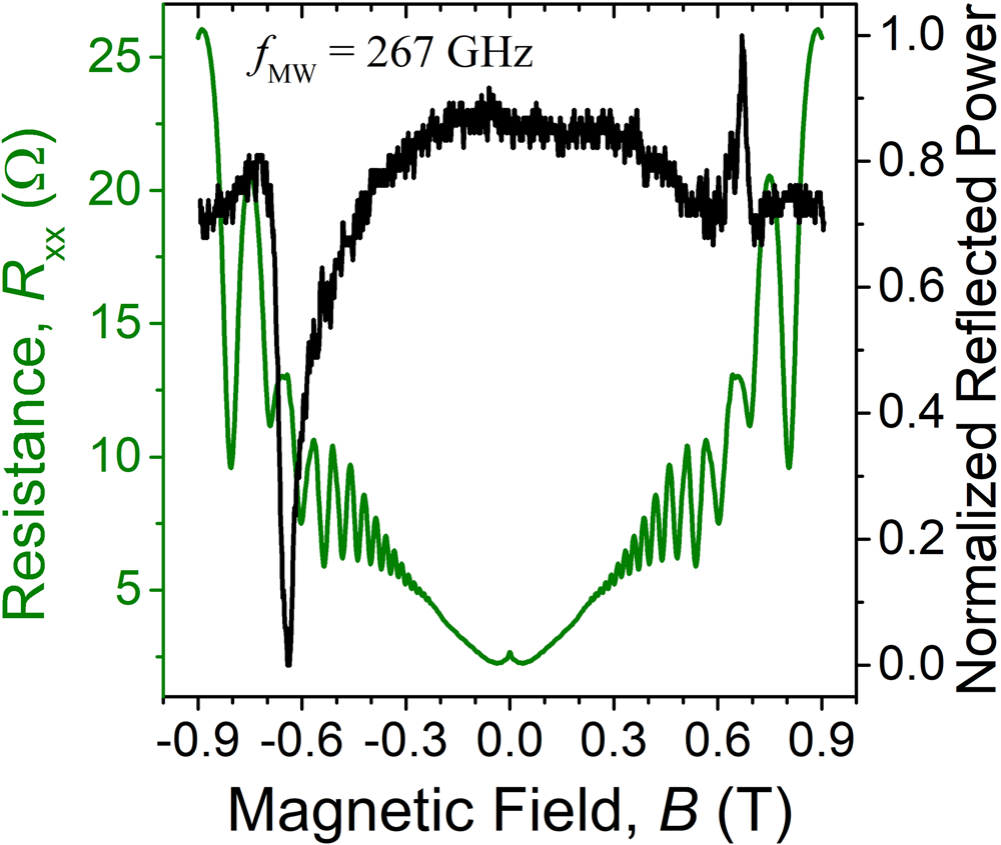
Reflected power (right ordinate) and the diagonal resistance, *R*_*xx*_, (left ordinate) as a function of magnetic field, *B*, measured in a GaAs/AlGaAs 2DES heterostructure at temperature *T* = 1.7 K and frequency *f* = 267 GHz. The coexisting resonances seen in both signals, peaks in reflected power at |*B*| = 0.0654 ± 0.015 T, and the change to SdH oscillations between |*B*| ≈ 0.60 T and |*B*| ≈ 0.70 T, point out the same underlying physical phenomenon: cyclotron or magnetoplasmon resonance.

In addition to measuring the reflected power signal, we concurrently measured the diagonal resistance, *R*_*xx*_, in our samples. Figure 5 plots the normalized reflected power (right ordinate) along with *R*_*xx*_ (left ordinate) as a function of magnetic field at microwave frequency, *f* = 267 GHz. Above |*B*| = 0.3, the resistance signal shows regular SdH oscillations, with apparent reduction in oscillations between |*B*| ≈ 0.60 T and |*B*| ≈ 0.70 T. These disturbances in SdH oscillation amplitude were observed only under irradiation, suggesting that the location of changes in oscillations point to the location of the cyclotron resonance^6^. We also notice in Fig. 5, that the disturbance in SdH oscillations coincides with the location of the resonance peaks at |*B*| = 0.654 ± 0.015 T in the reflected power signal.

**Figure 6:**
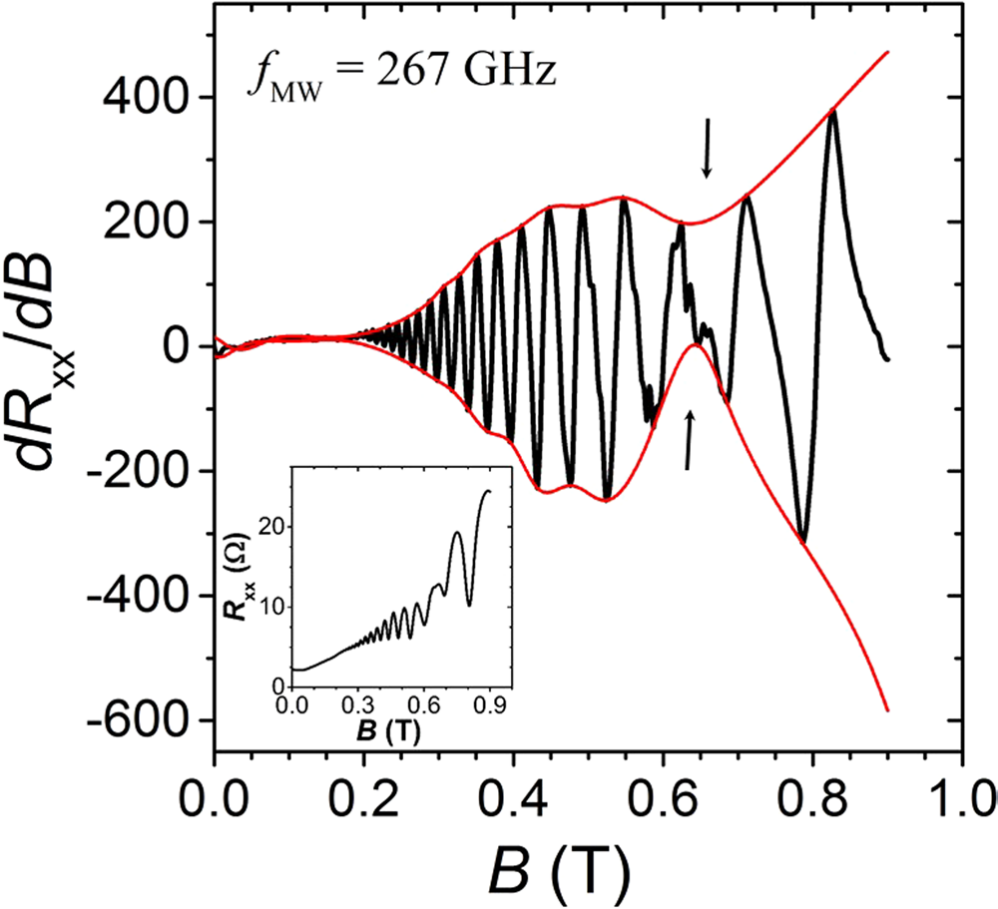
Derivative of diagonal resistance, *dR*_*xx*_/*dB*, as a function of magnetic field *B* in a GaAs/AlGaAs 2DES heterostructure at temperature *T* = 1.7 K and frequency *f* = 267 GHz. Red lines show envelopes drawn to the upper and lower bounds of *dR*_*xx*_/*dB*. Arrows point to the strongest change to SdH oscillations at *B* = 0.640 ± 0.005 T, found as an average of the upper envelope minimum at 0.636 T and the lower envelope maximum at 0.643 T. Inset: resistance signal, *R*_*xx*_, averaged over the positive and negative sides of the magnetic field, before calculating the derivative of the diagonal resistance, *dR*_*xx*_/*dB* in the main graph.

Locating resonant heating in SdH oscillations with high accuracy can be difficult, as they can spread over a wide span of magnetic fields. We circumvent this problem by calculating the derivative of diagonal resistance, *dR*_*xx*_/*dB*, and plotting it as a function of magnetic field, as shown in Fig. 6. Drawing the upper and lower envelopes (red line) to the boundaries of *dR*_*xx*_/*dB* then aids in pinpointing the strongest change to the SdH oscillations, i.e. the average magnetic field location of the upper minimum and lower maximum envelope (|*B*| = 0.640 ± 0.005 T in Fig. 6). The shallower extrema on the upper and lower envelopes, near 0.47 T in Fig. 6, are not investigated in this report.

**Figure 7:**
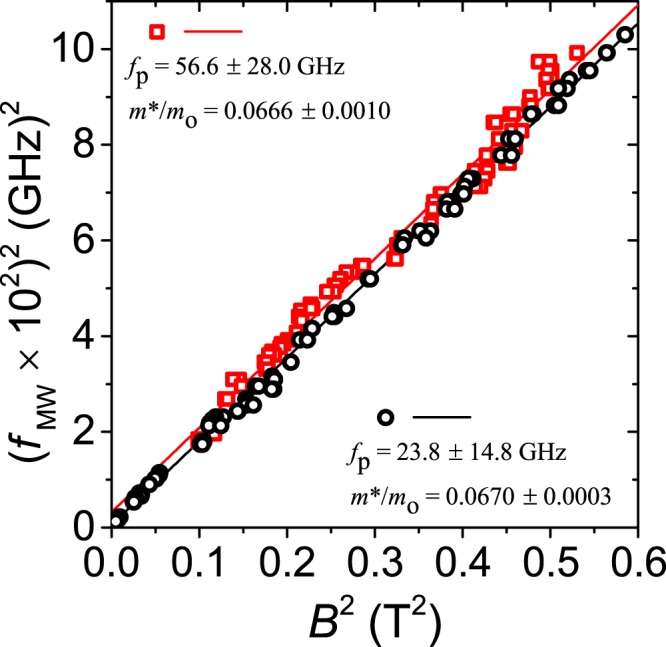
Comparison of resonance peak locations in reflected power (black circles, data also shown in Fig. 4) and in Shubnikov de Haas oscillations in the resistance signal (red squares), plotted on quadratic scale of *f*^2^ vs. *B*^2^. Data were measured concurrently in a sample with density *n*_*s*_ ≈ 2.4 × 10^11^ cm^−2^. Effective mass ratio, m*/*m*_*o*_, and plasmon frequency, *f*_*p*_, found from the slope and intercept, respectively, are indicated within the plot.

Using the method shown in Fig. 6, we locate the resonance positions in SdH oscillations over the measured frequency range, and plot them in Fig. 7 (red squares), in a quadratic scale of *f*^2^ vs. *B*^2^. Effective mass ratio, m*/*m*_*o*_ = 0.0666 ± 0.0010 and plasmon frequency, *f*_*p*_ = 56.6 ± 28.0 GHz, are found from slope and intercept of linear fit through data, respectively. These values agree with *f*_*p*_ = 54.0 ± 30.0 GHz and m*/*m*_*o*_ = 0.0681 ± 0.0010 found from resonance positions in SdH oscillations for the second sample, with *n*_*s*_ ≈ 3.3 × 10^11^ cm^−2^ (data not shown). For comparison, the resonance positions in the reflected power, measured in the same sample, are also shown in Fig. 7 (black circles). It is clear that both signals show resonance in the same place. This implies that both phenomena, though measured via different methods, point to the same physics in the sample, i.e., cyclotron or magnetoplasmon resonance. The higher error in *f*_*p*_ and m* values estimated from the data in SdH oscillations relates to the fact that SdH oscillations could not be measured below 0.3 T and to the overall higher inaccuracy in locating the resonance positions in SdH.

## Discussion and Summary

At liquid helium temperatures, we have measured microwave reflection and standard diagonal magnetoresistance as a function of the magnetic field in high mobility GaAs/AlGaAs heterostructures under linearly polarized photoexcitation over the wide frequency band spanning from 30 to 330 GHz. The microwave reflection indicated a cyclotron or magnetoplasmon resonance that shifted to higher magnetic fields with increasing frequency of photo-excitation on both sides of the magnetic field axis. The diagonal magnetoresistance exhibited Shubnikov-de Haas oscilllations with a reduction in the SdH amplitude in the vicinity of the cyclotron or magnetoplasmon resonance. This feature is attributed to resonant electron heating in the vicinity of the resonance and an associated reduction in the SdH amplitude. The magnetoplasma effect was further investigated by plotting *f*^2^ vs. *B*^2^. The results suggested a finite frequency zero-magnetic-field intercept, providing an estimate for the plasma frequency. The experimentally measured plasma frequency was somewhat lower than the estimated plasma frequency for these Hall bars. The results, in sum, are consistent with an effective mass ratio of m^*^/*m* = 0.067, the standard value, even in these high mobility GaAs/AlGaAs devices. Thus, the results show the possibility of observing the cyclotron or magnetoplasmon resonance from the reflected excitation and also in the Shubnikov-de Haas magnetoresistance oscillations in the photoexcited high mobility 2D electron specimen.

## Methods

GaAs/AlGaAs heterostructures were patterned into Hall bar devices with a width *W* = 200 *μ*m by photolithography. These devices were loaded onto a waveguide sample holder and inserted into a liquid helium cryostat to place the specimen at the center of a superconducting solenoid magnet. The samples were immersed in liquid helium for all the reported measurements. Commercially available power sensors were attached to one arm of a waveguide coupler, see Fig. 1, to capture the reflected power from the specimen, for the reflection measurements as a function of the magnetic field. The reflected power was read-out by a power meter. Note that, since the reflected resonance signal was significantly weaker than the incident photo-excitation, it was necessary to remove via an offset the background reflection, to realize the necessary enhanced sensitivity to observe cyclotron or magnetoplasmon resonance in the reflected signal. Magnetotransport measurements were performed, concurrently with the reflection measurements, using low frequency lock-in techniques, in order to obtain the diagonal resistance *R*_*xx*_ vs. the magnetic field. Measurements are reported here for a pair of samples which exhibited electron density *n*_*s*_ = 2.4 × 10^11^ cm^−2^ and mobility *μ* = 10 × 10^6^ cm^2^/Vs for the first sample and *n*_*s*_ = 3.3 × 10^11^ cm^−2^ and *μ* = 6 × 10^6^ cm^2^/Vs for the second sample.

The linearly polarized photo-excitation was produced using a number of sources: Over the 30–50 GHz frequency band, the photoexcitation was generated using commercially available synthesizers and conveyed via semirigid coax to an electric dipole launcher atop the waveguide. A 6x multiplier mm-wave module served to provide radiation between 65–110 GHz. Above 110 GHz, a highly stable and frequency tunable microwave oscillator in the 10–20 GHz region, with a linewidth of 1 Hz and output power of 17 dBm, excited a series of voltage biased- and unbiased- frequency multipliers. The frequency multiplication stage immediately following the 10–20 GHz oscillator was a frequency-doubling Spacek amplifier. Subsequent frequency multipliers, based on a planar GaAs Schottky diode technology have individual multiplication factors of ×2 or ×3 and are daisy-chained to obtain the desired output frequency up to 330 GHz. At the highest frequencies, attenuation between the radiation source and specimen is estimated to be between 10 and 20 dB, caused by a number of factors including mode mismatch, Ohmic losses, and atmospheric attenuation at the highest frequencies.

## References

[1] Ando T, Fowler AB, Stern F (1982). Electronic properties of two-dimensional systems. Rev. Mod. Phys..

[2] Kriisa A (2017). Remotely sensed microwave reflection in the microwave irradiated GaAs/AlGaAs two-dimensional electron system. J. Phys. Conf. Ser..

[3] Mani RG (2002). Zero-resistance states induced by electromagnetic wave excitation in GaAs/AlGaAs heterostructures. Nature.

[4] Zudov MA, Du RR, Pfeiffer LN, West KW (2003). Evidence for a new dissipationless effect in 2D electronic transport. Phys. Rev. Lett..

[5] Mani, R. G. *et al*. Demonstration of a 1/4 cycle phase shift in the radiation-induced oscillatorymagnetoresistance in GaAs/AlGaAs devices. *Phys. Rev. Lett*. **92**, 146801-1-4 (2004). 10.1103/PhysRevLett.92.146801 15089564

[6] Kovalev AE, Zvyagin SA, Bowers CR, Reno JL, Simmons JA (2004). Observation of a node in the quantum oscillations induced by microwave radiation. Sol. St. Comm..

[7] Mani, R. G. *et al*. Radiation induced oscillatory Hall effect in high mobility GaAs/AlGaAs devices. *Phys. Rev. B* **69**, 161306-1-4 (2004).

[8] Mani, R. G. *et al*. Radiation induced zero-resistance states in GaAs/AlGaAs heterostructures: Voltagecurrent characteristics and intensity dependence at the resistance minima. *Phys. Rev. B* **70**, 155310-1-5 (2004).

[9] Mani, R. G. *et al*. Radiation-induced oscillatory magnetoresistance as a sensitive probe of the zero-field spin splitting in high-mobility GaAs/AlGaAs devices. *Phys. Rev. B* **69**, 193304-1-4 (2004).

[10] Mani RG (2004). Zero-resistance states induced by electromagnetic-wave excitation in GaAs/AlGaAs heterostructures. Physica E (Amsterdam).

[11] Mani RG (2004). Radiation-induced zero-resistance states with resolved Landau levels. Appl. Phys. Lett..

[12] Simovic, B., Ellenberger, C., Ensslin, K. & Wegscheider, W. Density dependence of microwave induced magnetoresistance oscillations in a two-dimensional electron gas. *Phys. Rev. B* **71**, 233303-1-4 (2005).

[13] Mani, R. G. Radiation-induced oscillatory magnetoresistance in a tilted magnetic field in GaAs/AlGaAs devices. *Phys. Rev. B* **72**, 075327-1-5 (2005). 10.1103/PhysRevLett.92.146801 15089564

[14] Mani RG (2004). Photo-excited zero-resistance states in quasi-two-dimensional GaAs/AlGaAs devices. Sol. St. Comm..

[15] Smet, J. H. *et al*. Circular-polarization-dependent study of the microwave photoconductivity in a two-dimensional electron system. *Phys. Rev. Lett*. **95**, 116804-1-4 (2005). 10.1103/PhysRevLett.95.116804 16197030

[16] Mani RG (2005). Spin characterization and control over the regime of the radiation-induced zero-resistance states. IEEE Trans. Nanotechnol..

[17] Mani, R. G. Radiation-induced decay of Shubnikov-de Haas oscillations in the regime of the radiationinduced zero-resistance states. *Appl. Phys. Lett*. **91**, 132103-1-3 (2007).

[18] Wirthmann, A. *et al*. Far-infrared-induced magnetoresistance oscillations in GaAs/AlGaAs-based two-dimensional electron systems. *Phys. Rev. B* **76**, 195315-1-5 (2007).

[19] Studenikin, S. A. *et al*. Frequency quenching of microwave-induced resistance oscillations in a high-mobility two-dimensional electron gas. *Phys. Rev. B* **76**, 165321-1-6 (2007).

[20] Mani, R. G. Narrow-band radiation-sensing in the Terahertz and microwave bands using the radiationinduced magnetoresistance oscillations. *Appl. Phys. Lett*. **92**, 102107-1-3 (2008).

[21] Wiedmann, S. *et al*. Interference oscillations of microwave photoresistance in double quantum wells. *Phys. Rev. B* **78**, 121301-1-4 (2008).

[22] Mani, R. G., Johnson, W. B., Umansky, V., Narayanamurti, V. & Ploog, K. Phase study of oscillatory resistances in microwave irradiated and dark GaAs/AlGaAs devices: Indications of an unfamiliar class of integral quantum Hall effect. *Phys. Rev. B* **79**, 205320-1-10 (2009).

[23] Chepelianskii, A. D. & Shepelyansky, D. L. Microwave stabilization of edge transport and zeroresistance states. *Phys. Rev. B* **80**, 241308-1-4 (2009).

[24] Wiedmann, S. *et al*. Magnetoresistance oscillations in multilayer systems: Triple quantum wells. *Phys. Rev. B* **80**, 245306-1-9 (2009).

[25] Konstantinov, D. & Kono, K. Photon-induced vanishing of magnetoconductance in 2D electrons on liquid helium. *Phys. Rev. Lett*. **105**, 226801-1-4 (2010). 10.1103/PhysRevLett.105.226801 21231410

[26] Mani, R. G., Gerl, C., Schmult, S., Wegscheider, W. & Umansky, V. Nonlinear growth with the microwave intensity in the radiation-induced magnetoresistance oscillations. *Phys. Rev. B* **81**, 125320-1-6 (2010).

[27] Wiedmann, S., Gusev, G. M., Raichev, O. E., Bakarov, A. K. & Portal, J. C. Thermally activated intersubband scattering and oscillating magnetoresistance in quantum wells. *Phys. Rev. B* **82**, 165333-1-8 (2010).

[28] Wiedmann, S., Gusev, G. M., Raichev, O. E., Bakarov, A. K. & Portal, J. C. Microwave zero-resistance states in a bilayer electron system. *Phys. Rev. Lett*. **105**, 026804-1-4 (2010). 10.1103/PhysRevLett.105.026804 20867726

[29] Ramanayaka, A. N., Mani, R. G. & Wegscheider, W. Microwave induced electron heating in the regime of the radiation-induced magnetoresistance oscillations. *Phys. Rev. B* **83**, 165303-1-5 (2011).

[30] Mani, R. G., Ramanayaka, A. N. & Wegscheider, W. Observation of linear-polarization-sensitivity in the microwave-radiation-induced magnetoresistance oscillations. *Phys. Rev. B* **84**, 085308-1-4 (2011).

[31] Ramanayaka, A. N., Mani, R. G., Inarrea, J. & Wegscheider, W. Effect of rotation of the polarization of linearly polarized microwaves on the radiation-induced magnetoresistance oscillations. *Phys. Rev. B* **85**, 205315-1-6 (2012).

[32] Mani RG, Hankinson J, Berger C, de Heer WA (2012). Observation of resistively detected hole spin resonance and zero-field pseudo-spin splitting in graphene. Nature Commun..

[33] Konstantinov, D., Monarkha, Y. & Kono, K. Effect of coulomb interaction on microwave-induced magnetoconductivity oscillations of surface electrons on liquid helium. *Phys. Rev. Lett*. **111**, 266802-1-5 (2013). 10.1103/PhysRevLett.111.266802 24483809

[34] Mani RG, Kriisa A, Wegscheider W (2013). Magneto-transport characteristics of a 2D electron system driven to negative magneto-conductivity by microwave photoexcitation. Sci. Rep..

[35] Mani, R. G. *et al*. Terahertz photovoltaic detection of cyclotron resonance in the regime of the radiation-induced magnetoresistance oscillations. *Phys. Rev. B* **87**, 245308-1-8 (2013).

[36] Mani RG, Kriisa A, Wegscheider W (2013). Size-dependent giant-magnetoresistance in millimetre scale GaAs/AlGaAs 2D electron devices. Sci. Rep..

[37] Mani RG, von Klitzing K, Ploog K (1993). Magnetoresistance over the intermediate localization regime in GaAs/AlGaAs quantum wires. Phys. Rev. B.

[38] Ye, T., Liu, H-C.,Wegscheider,W. & Mani, R. G. Combined study of microwave-power/linear polarization dependence of the microwave-radiation-induced magnetoresistance oscillations in GaAs/AlGaAs devices. *Phys. Rev. B* **89**, 155307-1-5 (2014).

[39] Chepelianskii AD, Watanabe N, Nasyedkin K, Kono K, Konstantinov D (2015). An incompressible state of a photo-excited electron gas. Nat. Comm..

[40] Ye T, Liu H-C, Wang Z, Wegscheider W, Mani RG (2015). Comparative study of microwave radiationinduced magnetoresistive oscillations induced by circularly- and linearly- polarized photoexcitation. Sci. Rep..

[41] Mani RG (2016). Method for determining the residual electron- and hole- densities about the neutrality point over the gate-controlled n-p transition in graphene. Appl. Phys. Lett..

[42] Herrmann, T. *et al*. Analog of microwave-induced resistance oscillations induced in GaAs heterostructures by terahertz radiation. *Phys. Rev. B* **94** 081301-1-5 (2016).

[43] Liu, H-C., Samaraweera, R. L., Reichl, C., Wegscheider, W. & Mani, R. G. Study of the angular phase shift in the polarization angle dependence of the microwave induced magnetoresistance oscillations. *Phys. Rev. B* **94**, 245312-1-7 (2016).

[44] Shi Q (2015). Shubnikov de Haas oscillations in a two-dimensional electron gas under subterahertz radiation. Phys. Rev. B.

[45] Wang Z, Samaraweera RL, Reichl C, Wegscheider W, Mani RG (2016). Tunable electron heating induced giant magnetoresistance in the high mobility GaAs/AlGaAs 2D electron system. Sci. Rep..

[46] Samaraweera RL (2017). Mutual influence between current-induced giant magnetoresistance and radiation-induced magnetoresistance oscillations in the GaAs/AlGaAs 2DES. Sci. Rep..

[47] Liu H-C, Reichl C, Wegscheider W, Mani RG (2018). B-periodic oscillations in the Hall resistance induced by a dc-current bias under combined microwave excitation and dc-current bias in the GaAs/AlGaAs 2D system. Scientific Reports.

[48] Samaraweera RL (2018). Coherent backscattering in the quasi-ballistic ultra-high mobility GaAs/AlGaAs 2DES. Scientific Reports.

[49] Munasinghe CR (2018). Electron heating induced by an ac-bias current in the regime of Shubnikov-de Haas oscillations in the high mobility GaAs/AlGaAs two-dimensional electron system. J Phys Condens Matter.

[50] Nanayakkara TR (2018). Electron heating induced by microwave photoexcitation in the GaAs/AlGaAs two-dimensional electron system. Phys. Rev. B..

[51] Vasiliadou E (1993). Collective response in the microwave photoconductivity of Hall bar structures. Phys. Rev. B.

[52] Mani RG, von Klitzing K (1992). Localization at high magnetic fields in GaAs/AlGaAs. Phys. Rev. B.

[53] Durst, A. C., Sachdev, S., Read, N. & Girvin, S. M. Radiation-induced magnetoresistance oscillations in a 2D electron gas. *Phys. Rev. Lett*. **91**, 086803-1-4 (2003). 10.1103/PhysRevLett.91.086803 14525267

[54] Ryzhii V, Suris R (2003). Nonlinear effects in microwave photoconductivity of two-dimensional electron systems. J. Phys.: Cond. Matt..

[55] Andreev, A. V., Aleiner, I. L. & Millis, A. J. Dynamical symmetry breaking as the origin of the zero-dc-resistance state in an ac-driven system. *Phys. Rev. Lett*. **91**, 056803-1-4 (2003). 10.1103/PhysRevLett.91.056803 12906622

[56] Koulakov, A. A. & Raikh, M. E. Classical model for the negative dc conductivity of ac-driven two-dimensional electrons near the cyclotron resonance. *Phys. Rev. B* **68**, 115324-1-4 (2003).

[57] Lei, X. L. & Liu, S. Y. Radiation-induced magnetoresistance oscillation in a two-dimensional electron gas in Faraday geometry. *Phys. Rev. Lett*. **91**, 226805-1-4 (2003). 10.1103/PhysRevLett.91.226805 14683265

[58] Rivera, P. H. & Schulz, P. A. Radiation-induced zero-resistance states: Possible dressed electronic structure effects. *Phys. Rev. B* **70**, 075314-1-6 (2004).

[59] Mikhailov SA (2004). Microwave-induced magnetotransport phenomena in two-dimensional electron systems: Importance of electrodynamic effects. Phys. Rev. B..

[60] Dmitriev, I. A., Vavilov, M. G., Aleiner, I. L., Mirlin, A. D. & Polyakov, D. G. Theory of microwaveinduced oscillations in the magnetoconductivity of a two-dimensional electron gas. *Phys. Rev. B* **71**, 115316-1-11 (2005).

[61] Torres, M. & Kunold, A. Kubo formula for Floquet states and photoconductivity oscillations in a two-dimensional electron gas. *Phys. Rev. B* **71**, 115313-1-13 (2005).

[62] Lei, X. L. & Liu, S. Y. Radiation-induced magnetotransport in high mobility two-dimensional systems: Role of electron heating. *Phys. Rev. B* **72**, 075345-1-10 (2005).

[63] Inarrea, J. & Platero, G. Theoretical approach to microwave radiation-induced zero-resistance states in 2D electron systems. *Phys. Rev. Lett*. **94**, 016806-1-4 (2005). 10.1103/PhysRevLett.94.016806 15698116

[64] Inarrea, J. & Platero, G. Temperature effects on microwave-induced resistivity oscillations and zero-resistance states in two-dimensional electron systems. *Phys. Rev. B* **72**, 193414-1-4 (2005).

[65] Raichev, O. E. Magnetic oscillations of resistivity and absorption of radiation in quantum wells with two populated subbands. *Phys. Rev. B* **78**, 125304-1-14 (2008).

[66] Inarrea, J. Effect of frequency and temperature on microwave-induced magnetoresistance oscillations in two-dimensional electron systems. *Appl. Phys. lett*. **92**, 192113-1-3 (2008).

[67] Dmitriev, I. A., Khodas, M., Mirlin, A. D., Polyakov, D. G. & Vavilov, M. G. Mechanisms of the microwave photoconductivity in two-dimensional electron systems with mixed disorder. *Phys. Rev. B* **80**, 165327-1-9 (2009).

[68] Inarrea, J., Mani, R. G. & Wegscheider, W. Sublinear radiation power dependence of photoexcited resistance oscillations in two-dimensional electron systems. *Phys. Rev. B* **82**, 205321-1-5 (2010).

[69] Mikhailov, S. A. Theory of microwave-induced zero-resistance states in two-dimensional electron systems. *Phys. Rev. B* **83**, 155303-1-12 (2011).

[70] Inarrea, J. Influence of linearly polarized radiation on magnetoresistance in irradiated two-dimensional electron systems. *Appl. Phys. Lett*. **100**, 242103-1-3 (2012).

[71] Lei X. L. & Liu, S. Y. Linear polarization dependence of microwave-induced magnetoresistance oscillations in high mobility two-dimensional systems. *Phys. Rev. B* **86**, 205303-1-5 (2012).

[72] Iñarrea, J. Linear polarization sensitivity of magnetotransport in irradiated two-dimensional electron systems. *J. Appl. Phys*. **113**, 183717-1-5 (2013).

[73] Zhirov, O. V., Chepelianskii, A. D. & Shepelyansky, D. L. Towards a synchronization theory of microwave-induced zero-resistance states. *Phys. Rev. B* **88**, 035410-1-14 (2013).

[74] Raichev, O. E. Theory of magnetothermoelectric phenomena in high-mobility two-dimensional electron systems under microwave irradiation. *Phys. Rev. B* **91**, 235307-1-16 (2015).

[75] Beltukov, Y. M. & Dyakonov, M. I. Microwave-induced resistance oscillations as a classical memory effect. *Phys. Rev. Lett*. **116**, 176801-1-5 (2016). 10.1103/PhysRevLett.116.176801 27176530

[76] Chang C-C, Chen G-Y, Lin L (2016). Dressed photon induced resistance oscillation and zero-resistance in arrayed simple harmonic oscillators with no impurity. Sci. Rep..

[77] Miura, N. *Physics of Semiconductors in High Magnetic Fields* (New York, Oxford University Press, 2007).

[78] Kohn W (1961). Cyclotron resonance and de Haas-van Alphen oscillations of an interacting electron gas. Phys. Rev..

[79] Merkt U (1996). Cyclotron resonance of localized electron systems in the magnetic quantum limit. Phys. Rev. Lett..

[80] Fano U (1961). Effects of configuration interaction on intensities and phase shifts. Phys. Rev..

[81] Mani RG, Anderson JR (1988). Study of the single particle and the transport lifetimes in GaAs/AlGaAs. Phys. Rev. B.

